# Where a psychopathic personality matters at work: a cross-industry study of the relation of dark triad and psychological capital

**DOI:** 10.1186/s40359-023-01266-4

**Published:** 2023-08-17

**Authors:** Birgit Stephan, Dominik Lechner, Mariella Stockkamp, Matthias F. C. Hudecek, Dieter Frey, Eva Lermer

**Affiliations:** 1 Center for Leadership and People Management, LMU Munich, Geschwister-Scholl-Platz 1, 80539 Munich, Germany; 2 Department of Experimental Psychology, University of Regensburg, Universitätsstraße 31, 93053 Regensburg, Germany; 3 Department of Business Psychology, Technical University of Applied Sciences Augsburg, An der Hochschule 1, 86161 Augsburg, Germany

**Keywords:** Narcissism, Psychological Capital, Dark Triad, Gender differences, person-environment fit, Homogeneity Hypothesis

## Abstract

**Background:**

The concepts of Dark Triad and Psychological Capital (PsyCap) have been extensively researched separately, but until one recent study, their interrelation has not been investigated. Purpose of this study was to uncover differences of the relationship of both concepts across work related industries.

**Methods:**

In total, 2,109 German employees across 11 industries completed a questionnaire on Dark Triad (narcissism, psychopathy and Machiavellianism) and PsyCap. Multiple regression analyses were used to test the association of both concepts across industries.

**Results:**

Values of narcissism, psychopathy and PsyCap generally differed between industries. No significant differences were found for Machiavellianism. While narcissism relates positively to PsyCap in all industry sectors, psychopathy only showed a negative relation to PsyCap in some sectors. For industries architecture, automotive and consulting, psychopathy did not significantly predict PsyCap.

**Conclusions:**

We argue that different expectations of employees per industry make it easier or harder for different personalities to assimilate (homogeneity hypothesis) to the work context (measured by PsyCap). Future studies should investigate this further with other variables such as person-organization-fit. This study was, however, the first to simultaneously investigate Dark Triad and PsyCap among employees and their respective industry. It extends previous findings by revealing differences of both concepts across and within industry sectors. The study can help to reconsider in which industries Dark Triad personality affects PsyCap as antecedent of workplace outcomes such as work satisfaction or job performance.

**Supplementary Information:**

The online version contains supplementary material available at 10.1186/s40359-023-01266-4.

## Introduction

In the past two decades, the concepts *Psychological Capital* (PsyCap, [1]) and *Dark Triad* (DT, [2]) have received much attention in research. PsyCap is understood as a positive psychological resource and is made up of four measurable factors that are seen as conceptually independent: *hope, self-efficacy*, *resilience*, and *optimism* (HERO, [1]). Research on the (positive) influence of PsyCap on workplace outcomes such as work satisfaction (e.g., [3, 4]), commitment [5], psychological well-being [4, 6, 7], organizational citizenship behavior [8, 9], and performance [3, 10, 11] has been fairly sufficient in the past years, as meta-analyses show (e.g., [12]). DT is composed of three facets: narcissism, psychopathy and Machiavellianism. These are the most investigated socially aversive personality traits in psychological science [2]. Research streams on DT range from its relation to other personality measurements such as the Big Five [13] to the organizational context with its negative and positive influence on workplace outcomes such as counterproductive workplace behaviors [14–16] or personal career [17–19].

### The current study

Until the recent study of Zhu and Geng [20], both concepts had not been studied together. Zhu and Geng [20] found in the 10-months longitudinal study that psychopathy and Machiavellianism were negatively related to PsyCap, whereas narcissism positively related to PsyCap. They were able to show that both PsyCap as well as DT facets together with sadism (known as “Dark Tetrad” [21]) remain stable across time while the relationship was shown to be a causal one with narcissism, psychopathy and Machiavellianism being causal antecedents of PsyCap and sadism being a consequence of PsyCap. This study along with most others in this context have neglected the work environment, especially the industry in which participants work. As a result, little is known about whether and, if so, how far the expressions of DT and PsyCap differ across different industries. Until today, only few studies have analyzed the differences of personality, and especially DT and PsyCap between industries (see review of Stephan et al., unpublished manuscript). According to Stephan et al., studies on industry differences and DT can be split into three ways of measuring which DT personalities are prevalent among various industries: 1) DT personalities are based on what participants expect in industry sectors (e.g., [22]), 2) DT personalities are based on what participants have experienced themselves in industry sectors (e.g., [23]), and 3) actual self-assessed DT personalities by industry sector [24, 25]. The latter two studies are the few that examine actual prevalence of DT facets in different industries, however, they each only take one of the three DT facet into account. To the best of the authors’ knowledge, no comparison of all three DT facets between more than two industry sectors has been done based on people’s actual DT personalities. As of PsyCap, no study is known that looks at differences across industries. The goal of this study is thus to investigate A) the prevalence of differences in DT and PsyCap between industries (hypothesis 1 and 2), and B) how the relationship between both concepts varies between industries (research question).

Basis of this research is to combine existing research results with a theoretic basis that can explain personality differences between industry sectors. Research has confirmed that different industries attract different kinds of personality (e.g., [26]). Schneider et al. [27] showed a significant effect of the industry on personality in a large cross-industry sample – referred to as *homogeneity hypothesis*. This effect can be explained by people actively choosing the company that matches their need structure best [28, 29], and more downstream the organization helping new employees to assimilate with industry values [30, 31]. Further, employees whose personality do not fit to what is required in their organization leave [32]. The result is a congruence of personality and organizational setting which is reflected in DT facets. Differences in DT across industries have so far only been identified for psychopathic traits and narcissism [24, 25].

As for psychopathy, a study by Boddy [23] found higher prevalence of psychopathic personalities as experienced by participants working in the finance and insurance sector as well as governmental and educational sector (around 28% of participants had experienced psychopathic behavior in both industries). In comparison, lower prevalence was found in retail (16%), agriculture & manufacturing (19%) and health service (24%). Clarke [33] gives an explanation with psychopathic personalities tending to be more focused on themselves, excluding others and trying to get into a position of power and prestige. To achieve this, they tend to not shy away from risk-taking. This makes them attracted to industries that either involve structures of power or money or relate to more opportunities of risk-taking [34]. The finance and insurance sector provides opportunities to gain money, wealth or prestige and is associated with more risk-related domains, whereas governmental and educational sector is known to consist of structures of power [23, 35, 36]. Accordingly, both industries provide an environment that fits psychopathic personalities which could attract employees with higher psychopathic traits. As for narcissism, there are no known differences across industries per se. However, narcissism is known to be more frequent among men than women as a meta-analysis of Grijalva et al. [37] showed. In combination with this, industries differ in the distribution of gender with higher shares of male employees prevalent in certain industries (e.g., IT or automotive, [38]). Grijalva et al. [37] showed in their review of 355 studies that gender differences can lead to different magnitude of narcissism for some sub-facets (namely on the dimensions exploitative behavior and authority) whereas no or only small effects were found for other sub-facets of narcissism (namely grandiose and vulnerable, additional results from [39]). This gender difference remained consistent over time as well as stable across different age groups. Considering both perspectives (distribution of gender per industry and gender differences for Narcissism), we expect those industry sectors that employ more men than women to show a higher prevalence of narcissism, specifically IT, automotive, finance and insurance, and architecture and construction [40]. While so far, these industry differences in narcissism have been explained by gender, the *homogeneity hypothesis* [27] provides an additional explanation as to why narcissistic personalities feel more attracted by one or the other industry – namely, a match of personality and expectations within the industry as an explanatory variable in this suggesting that people with higher narcissism tend to choose domains that expect or (unconsciously) encourage narcissistic behavior. Closing with Machiavellianism, no study is known among the authors that reports industry differences. Despite missing research, we propose to detect industry differences also for Machiavellianism which would be in line with the *homogeneity hypothesis* suggesting people to join industries that fit their need structure which is influenced by their personality. In total, previous research on DT in the context of industry differences is scarce and lacking an embracing theory to explain variations. Currently, differences are either explicated by gender (as for narcissism, [37, 41]) or contextual attributes of the industries (for psychopathy e.g., [23]). In this view, we expect the *homogeneity hypothesis* [27] to add a theoretical explanation to be applied for all three DT facets.

As mentioned before, to the best of the authors’ knowledge, there is no research on industry differences for PsyCap so far. However, in light of expected differences of DT facets between industries [23, 35, 36, 39] and a causal relation of DT on PsyCap [20], PsyCap should differ as well between industries. To explain this with an example: Assumed that people in the finance and insurance sector showed higher psychopathic personality traits than in other industries due to a better fit of this personality to this specific industry structure (as proposed by [23]). Also, assumed that psychopathy had a negative impact on PsyCap (e.g. [20]). The result of both assumptions would be lower scores of PsyCap among employees of the finance and insurance sector compared to other industries.

Lastly, with assumed differences of both DT and PsyCap between industries, there is a rising question how the relationship of these personality aspects (DT) and psychological resources (PsyCap) looks like in different industries. As of today, there is only evidence for the relationship based on a student sample [20], but not how a specific industry sector might enhance or attenuate this relation. Drawing on these previous findings, this study could provide an important contribution into how industries influence employees’ psychological resources which could subsequently impact organizational outcomes such as job satisfaction [3], organizational commitment [8], and mental health [42]. The following hypotheses and research question are proposed:

#### H1

DT facets a) narcissism, b) psychopathy, and c) Machiavellianism differ between industries.

#### H2

PsyCap differs between industries.

#### Research Question (RQ)

How does the relationship of DT facets and PsyCap change between industries?

## Method

### Participants and procedure

Participants for the online study were recruited via Facebook and via university platforms. The sample consisted of three groups: in parallel employed students of two German universities. The third group were employees or self-employed persons studying part-time recruited from a German university of applied sciences (with locations in 32 cities across Germany, [43]). Participants received course credits for their participation. After exclusion of incompletely filled questionnaires, participants with a participation duration outside of 1.5*interquartiles range were removed to prevent the inclusion of inattentively quick or long responses [44, 45]. The cleaned sample consisted of 2,109 participants. On average, participants were 27.7 (*SD* = 7.89) years old, and the majority was female (71.7%, 28.3% male). 47.7% of the sample had an A-level degree, 12.3% held a university degree. All participants were asked to indicate in which industry they worked based on a selection of 11 pre-defined industry options. 79.9% of participants successfully assigned themselves to a specific industry. Participants who could not assign themselves were asked to name their industry in an open field. With this approach, additional 6.6% of the sample could be later manually assigned to existing categories with two senior researchers working together to ensure multiple perspectives while decreasing personal bias as suggested by Corbin and Strauss [46]. Within the manual assignment of the “other” category, for example, participants who worked in real estate were assigned to sector architecture and construction, people working in publishing were assigned to retail and consumption (see supplementary material for further details). In total, 85.5% of participants could be allocated to a specific industry sector indicating a suitable categorization. Details of sample characteristics are shown in Table 1. Prior to the analysis of H1 and H2, participants from energy sector (N = 51) and telecommunication (N = 33) were excluded as they were well below the intended minimum sample size of 148 as calculated with a priori power analysis. Industries with a minimum of 100 participants were kept for the analysis following practices from previous research [47] but were tested carefully through a posteriori power analysis (see section “statistical analysis”). Participants of category “other” (N = 305) were further excluded due to a not identifiable industry affiliation, resulting in a final sample of 1,720 participants used for testing the hypothesis.

**Table 1:** Sample description

Variables	count (%)
Gender	
Male	596 (28.3%)
Female	1,513 (71.7%)
Education level	
A-level degree	1,005 (47.7%)
Apprenticeship	707 (33.5%)
Bachelor degree	129 (6.1%)
Master degree	109 (5.2%)
Doctorate degree	21 (1.0%)
Other or lower than A-level	138 (6.5%)
Industry	
Finance & insurance services	334 (15.8%)
Retail & consumption	280 (13.3%
Health care, medical & social services	222 (10.5%)
Public service, administration & transportation	215 (10.2%)
Automotive & engineering	160 (7.6%
Education & research	144 (6.8%)
Consulting	133 (6.3%)
IT	123 (5.8%)
Architecture & construction	109 (5.2%)
Energy	51 (2.4%)
Telecommunications	33 (1.6%)
Other/uncategorized	305 (14.5%)
Leadership Experience	
no	1,727 (81.9%)
yes	363 (17.2%)
NA	19 (0.9%)
Total	2,109 (100%)

### Measures

#### PsyCap

To assess Psychological Capital, the German adaption of the PsyCap Questionnaire (PCQ, [3]) was applied which comprises four subscales: self-efficacy [48], hope [49], optimism [50] and resilience [51]. Each of the subscales is measured by six items for which participants rated their level of agreement on a 6-point Likert scale (1 = strongly disagree, 6 = strongly agree). Items for each facet were averaged to create subscales. Cronbach’s α were .83 for *hope*, .84 for *self-efficacy*, .71 for *resilience* and .61 for *optimism*. Internal consistency for overall inventory PsyCap was .89.

#### Dark Triad

The German version of the Short Dark Triad (SD3, [39]) was used to measure individuals’ extent on the three DT facets. The scale consists of 27 items with nine items measuring each of the three personality facets. Participants were instructed to rate their extent of agreement to specific statements on a 5-point Likert scale (1 = *strongly disagree*, 5 = *strongly agree*). Items of each facet were averaged to create subscales with Cronbach’s α of .69 for *Narcissism*, .70 for *Psychopathy*, and .75 for *Machiavellianism*.

#### Industry sector

To determine affiliation to an industry sector, we provided participants a pre-defined selection which consisted of a combination of Boddy’s [23] categorization focusing on DT facets in each sector as well as Liu, Feils and Scholnick [52] who split industry field based on routineness and complexity. The industry sectors resulting from this combination aimed to be exhaustive yet not too granular to enable minimum sample sizes per sector. The industries provided match 70% of the industry categories used for the audience of university graduates [53] and thus can be seen as a representative set of industries for this study. Participants were shown these sectors and asked to select the industry in which they work themselves. An “other” option was additionally given which was later manually mapped to the existing industry sectors by the authors. Industry sectors and respective sample share are listed in Table 1.

#### Control variables

Gender, age, educational level [17, 18, 37, 54], and leadership experience [55, 56] were considered control variables as they have been shown to impact either DT or PsyCap. Thus, age (*open question*), education level, gender, and leadership role experience (*no, yes*) were measured. Descriptives are shown in Table 1.

### Statistical analysis

All analyses were conducted with R studio (version 1.2.5019). Multilevel analysis was considered to analyze differences of DT facets (H1) and PsyCap (H2) between industries since we expected variances of each of the two concepts to be nested in the industry sector. Final analysis was done via ANOVAs supplemented with Tukey posthoc tests. For the research question, several multiple regressions were performed to understand the relation of DT and PsyCap between industries better. Lastly, gender was considered as potential exploratory variable via a mediation model (following the approach of [57]) to understand if variances between industries are related to gender differences.

To determine the minimum sample size needed to test the hypothesis, a priori power analysis was performed. While previous studies found small to medium effect sizes [22, 25] comparing very different industries in terms of structure and work routine, this study was set up more conservatively expecting small effect sizes. Results indicated the need for a minimum of 148 participants per industry group to achieve 80% power (k = 11, f = 0.1, α = 0.05). Since this number was not reached for four of the groups used in the final sample (minimum n = 109), additional a posteriori power analysis was conducted resulting in an achieved power of 84% for ANOVA (k = 9, n = 109, f = 0.13, α = 0.05) and 95% for regression analysis (u = 4, v = 104, f2 = 0.15, α = 0.05).

## Results

Zero-order-correlations of DT facets and PsyCap, means and standard deviations are depicted in Table 2.

**Table 2:** Descriptive statistics, internal zero-order correlations (Spearman) for the relationship between DT facets and PsyCap

	*M*	*SD*	*1*	*2*	*3*	*4*
*1. Narcissism*	2.81	0.58	-			
*2. Psychopathy*	2.10	0.59	.39**	-		
*3. Machiavellianism*	3.04	0.62	.30**	.49**	-	
*4. PsyCap*	4.59	0.55	.33**	− .04	− .02	-

*Note. M* and *SD* are used to represent mean and standard deviation, respectively, *n* = 2,109.

* indicates *p* < .05, ** *p* < .01, *** *p* < .001

As first step, we followed a multilevel approach (see [58]) which was chosen to consider potential industry cluster effects sufficiently. Narcissism, psychopathy and Machiavellianism as well as PsyCap were measured as individual observations (level 1) which we assumed to be nested in industry sectors (level 2). To test the existence of a multilevel structure, intraclass correlations (ICCs) were calculated for an intercept-only model (model 1, Maximum-Likelihood estimation) with narcissism, psychopathy, Machiavellianism, and PsyCap as dependent variables each. ICCs in model 1 were 0.014 for narcissism, 0.013 for psychopathy, 0.004 for Machiavellianism, and 0.007 for PsyCap. This means that only 1.4% of variance in narcissism goes back to the industry sector as group variable. For psychopathy, Machiavellianism, and PsyCap, even less variance is explained by the industry. Following Musca et al. [59], the existent combination of number of individuals per group, number of groups and ICCs can lead to a Type I error percentage of around 20%, so Kish’s correction is needed if the multilevel approach is not followed through. With ICCs of less than 0.1, a multilevel approach is not needed for further analysis.

**Fig. 1:**
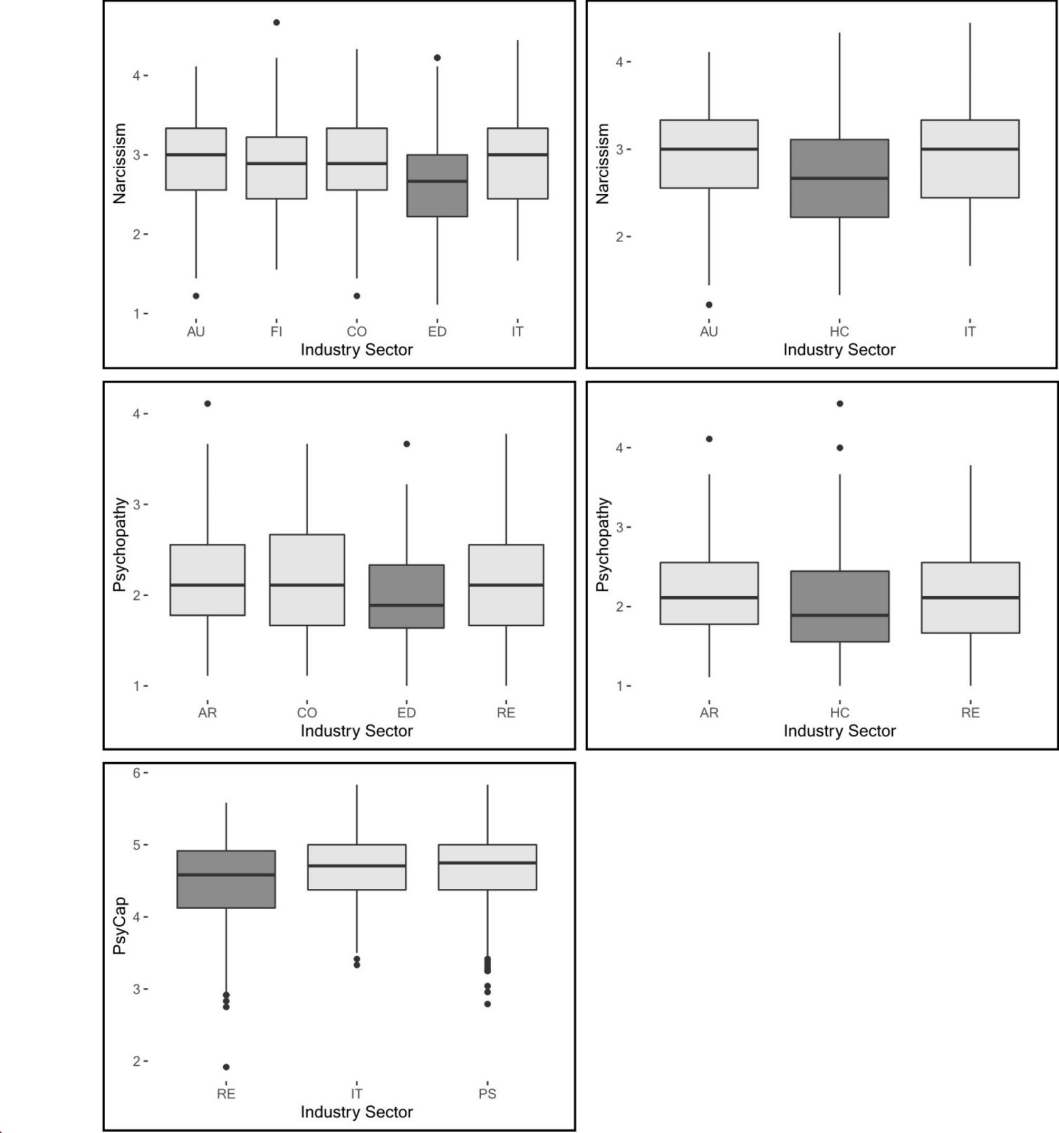
Tukey post hoc results representing differences in narcissism, psychopathy and PsyCap between industries *Note*. Box plot of significant post hoc analysis results. Industries with different colors in each plot differ significantly (min p <.1), industries in the same color with no significant difference *Abbreviations*: AR = Architecture & construction, AU = Automotive & Engineering, CO = Consulting, ED = Education & Research, FI = Finance & Insurance services, HC = Health Care, Medical and Social services, IT = IT, PS = Public Service, Administration & Transportation, RE = Retail & Consumption

As second step, differences between industries were analyzed with ANOVAs. Results show industry differences for narcissism (*F*(8, 1711) = 3.51, *p* < .001), psychopathy (*F*(8, 1711) = 3.21, *p* = .001), and PsyCap (*F*(8, 1711) = 2.33, *p* = .02), and no significant industry differences for Machiavellianism (*F*(8, 1711) = 1.61, *p* = .12; full details in Table 3). Since narcissism, psychopathy and PsyCap differ across industries, Tukey posthoc tests were run to identify which industry sectors show the highest pairwise differences in expression of DT facets (see Fig. 1 for differences in narcissism, psychopathy and PsyCap). Despite no significant differences found across all industries for Machiavellianism, posthoc tests were also run for this facet to check for single differences that were not discovered on an overall industry level.

**Table 3:** Differences in Narcissism, Psychopathy, Machiavellianism and PsyCap across industries.

		Measures			
		*Narcissism*	*Psychopathy*	*Machiavellianism*	*PsyCap*
Industry	Architecture & construction	2.86 (0.59)	2.24 (0.63)	3.06 (0.61)	4.65 (0.52)
	Automotive & engineering	2.91 (0.56)	2.05 (0.54)	3.09 (0.59)	4.63 (0.51)
	Finance & insurance services	2.85 (0.55)	2.11 (0.55)	3.05 (0.59)	4.59 (0.50)
	Consulting	2.88 (0.56)	2.18 (0.60)	3.04 (0.60)	4.56 (0.62)
	Education & research	2.67 (0.60)	1.98 (0.51)	3.02 (0.56)	4.57 (0.55)
	Health care, medical & social services	2.71 (0.60)	2.03 (0.59)	2.92 (0.64)	4.59 (0.57)
	Retail & consumption	2.81 (0.56)	2.06 (0.56)	3.10 (0.59)	4.48 (0.62)
	IT	2.90 (0.61)	2.07 (0.56)	3.00 (0.59)	4.67 (0.51)
	Public service, administration & transportation	2.78 (0.62)	2.07 (0.61)	3.05 (0.60)	4.64 (0.56)
	*df*	8	8	8	8
	*F* value	3.507	3.213	1.610	2.333
	*p* value	0.0005	0.0013	0.117	0.0172

*Note.* Means and standard variations (in brackets). Results of ANOVAs for each measure are shown in the bottom (including *df*, *F* value and *p* value); *n* = 1,720.

For narcissism (H1a), differences were found between education & research as industry with lowest values and automotive & engineering (*p* = .01), IT (*p* = .03), finance & insurance services (*p* = .05), and consulting (*p* = 0.05) with highest values (see Fig. 1). Additionally, pairwise differences in narcissism were found between health care & social services and automotive (*p* = .03) and no significant difference but approached significance with IT (*p* = .07). Narcissism scores were low for industry sectors education & research, and health care & social services, whereas high values were seen for automotive & engineering, finance & insurance services, consulting and IT. For psychopathy (H1b), differences were found between education & research with lowest values and architecture (*p* = .01), retail (*p* = .02), and with approached significance consulting (*p* = .09) with highest values. Further health care & social services differed in psychopathy traits compared to architecture (*p* = .05), and with approached significance retail (*p* = .09). Similar to narcissism, psychopathic values were low in health care and education sectors and high in architecture, consulting and retail. For Machiavellianism (H1c), differences were only found between health care & social services with lowest values and retail (*p* = .05) with highest values. For PsyCap (H2), differences were found between retail with lowest values and IT (*p* = .03) and public service (*p* = .04) with highest values. No other significant post hoc differences were found for PsyCap.

To ensure that differences between the industry sectors are not influenced by gender, we followed a bootstrapped mediation model ([57], bootstrap as used by [60]). This was the third step in the analysis.

As for narcissism (H1a), a positive unstandardized indirect effect of .07 (95% *CI* [0.001, 0.14], *p* < .05) was found which shows that narcissism mediates the effect of gender on industry sector. The direct effect was not significant (beta = -0.056, 95% *CI* [-0.36, 0.24], *p* = .70). This rules out the hypothesis that gender directly affects differences of narcissism in industries. For psychopathy (H1b), there was no significant indirect effect of 0.06 (95% *CI* [-0.05, 0.18], *p* = .30). Also, the direct effect of gender on industry sector was not significant (beta = -0.05, 95% *CI* [-0.38, 0.28], *p* = .75). Also, for PsyCap (H2), the bootstrapped mediation model revealed no significant unstandardized indirect effect of 0.01 (95% *CI* [-0.03, 0.05], *p* = .50). Additionally, the direct effect was not significant (beta = -0.004, 95% *CI* [-0.31, 0.30], *p* = .98).

Taken together, results thus support H1a and H1b indicating a difference between industries for narcissism and psychopathy. On the other side, results do not support H1c showing no significant difference of Machiavellianism between industries overall except from one difference between health care and retail industries. For narcissism, gender plays a role with a direct effect on narcissism trait resulting in narcissism being a mediator of gender on industry differences. While for psychopathy and PsyCap, no impact of gender was found. Differences between industries found for PsyCap support H2 and cannot be explained by gender.

Lastly, to test the research question on how the relationship between DT facets and PsyCap changes in different industries, several multiple regressions were performed (details of results in table 4). Prerequisite to conducting regression analysis is to check for collinearity. VIF values varied between 1.1 and 1.5 indicating a low to moderate collinearity between predictor variables, namely the DT facets. With a collinearity at this level, individual predictors do not need to be checked for an overestimation of effects [61]. As with testing hypotheses H1a-c and H2, for the research question a multilevel approach (see [58]) was considered first to take nesting of participants within industries into account. Due to ICCs below 0.1 for the predictor variables seen in the first part of the analysis, a multilevel approach is not needed. Thus, the data set was split into subsets for each industry sector and multiple regressions were run independently. To control the type I error rate, p-values were corrected using the Hommel method which is a bonferroni based method appropriate for independent hypothesis tests [62]. After correction, all effects stayed significant at least at the 5% level with only a few effects of control variables and one predictor to be lowered from 1% level to 5% level.

There are two distinct relational structures apparent among the industries: 1) Narcissism shows a positive effect on PsyCap (for sectors architecture (β = 0.34, *p* < .01, *R*^2^ = .13), automotive (β = 0.32, *p* < .001, *R*^2^ = .18), and consulting (β = 0.32, *p* < .05, *R*^2^ = .14), while there is no significant relation of psychopathy with PsyCap. 2) Along with a positive relation of narcissism comes a negative relation of psychopathy on PsyCap for sectors finance, education, health care, retail, IT and public service (results in table 4). Machiavellianism plays a minor role in predicting PsyCap from a statistical point of view.

**Table 4:** Regression results for Narcissism, Psychopathy, Machiavellianism on PsyCap for each industry sector

Variable	Architecture & construction		Automotive & engineering		Finance & insurance services		Consulting		Education & research		Health care, medical & social services		Retail & consumption		IT		Public service, administration & transportation	
	*B*	*SE*	*B*	*SE*	*B*	*SE*	*B*	*SE*	*B*	*SE*	*B*	*SE*	*B*	*SE*	*B*	*SE*	*B*	*SE*
Constant	3.66^**^	0.46	4.10^**^	0.38	3.79^**^	0.24	5.11^**^	0.52	3.83^**^	0.40	4.16^**^	0.31	3.95^**^	0.31	4.39^**^	0.37	4.21^**^	0.29
Narcissism	0.34^**^	0.08	0.32^**^	0.07	0.41^**^	0.05	0.32^*^	0.10	0.47^**^	0.07	0.33^**^	0.07	0.42^**^	0.07	0.33^**^	0.78	0.47^**^	0.06
Psychopathy	-0.09	0.10	-0.15	0.08	-0.14^*^	0.06	-0.17	0.10	-0.22^*^	0.09	-0.24^*^	0.08	-0.22^*^	0.07	-0.21^*^	0.09	-0.46^**^	0.07
Machiavellianism	-0.01	0.09	-0.12	0.07	-0.03	0.05	-0.21^*^	0.90	-0.06	0.08	0.01	0.07	-0.05	0.06	0.01	0.08	0.06	0.06
Leadership Experience^a^	0.22	0.11	0.34^*^	0.12	0.22^*^	0.08	0.09	0.13	0.06	0.11	0.15	0.09	0.21^*^	0.09	0.05	0.13	0.23^*^	0.08
Gender^b^	-0.01	0.11	-0.09	0.09	-0.11	0.06	-0.33^*^	0.13	0.01	0.10	-0.10	0.09	-0.17^*^	0.08	-0.23^*^	0.09	-0.21^*^	0.08
R^2^	0.13^**^		0.18^**^		0.20^**^		0.14^**^		0.22^**^		0.13^**^		0.16^**^		0.13^**^		0.36^**^	

*Note.* Multiple regressions run separately for participants belonging to each industry, p values corrected with Hommel’s method (1988). ^a^without leadership experience = 1; with leadership experience = 2. ^b^Male = 1, Female = 2.

*n* = 1,720.

^*^p < 0.05, ^**^p < 0.01.

## Discussion

Two major findings emerge from this research: 1) Differences between industry sectors were found in narcissism, psychopathy and PsyCap, while for Machiavellianism only one industry pair differed significantly (H1&H2). 2) The effect of DT facets on PsyCap changes between industries. For architecture, automotive and consulting sectors, only Narcissism relates to PsyCap while psychopathy does not. For all other sectors, both narcissism and psychopathy relate to PsyCap (research question).

### DT and PsyCap differ between industry sectors

We found several differences between industry sectors for narcissism, psychopathy as well as PsyCap, but only one for Machiavellianism. Psychopathic personalities were unequally distributed among the industries with higher values in architecture, consulting and retail and lower values in education and health care. Differences in psychopathy as well as PsyCap across industry sectors were not explained by gender. Results on psychopathy are in line with some assumptions such as a high prevalence in industries architecture and consulting due to higher risk needed in these sectors. On the other side, assumptions on a high prevalence in industry education and research were not confirmed. This indicates that psychopathic traits are more relevant in high-risk industries and less relevant when hierarchies or structures of (political) power are present [23]. In contrary to psychopathy, differences in narcissism across industry sectors could be explained by gender. Our sample revealed narcissism to mediate the relation of gender on industry sector and stands in contrast to the findings of Green, MacLean and Charles [63] who did not identify gender difference in (grandiose) narcissism. Difference in narcissism e.g. in sectors health care vs. automotive and health care vs. IT could thus be explained by a higher share of male participants (indirect effect). This is in line when looking at absolute scores whereas highest values of narcissism were prevalent in IT, automotive, architecture, finance and consulting.

### Two different structures found how DT facets and PsyCap are related

Results on the relation of DT to PsyCap are consistent to recent findings from Zhu and Geng [64] in regards to the positive relation of narcissism on PsyCap. Zhu and Geng found narcissism not only as consistent over time but as the one DT facet with a positive effect on psychological states and further even on physical health. Our study shows that the positive effect of narcissism on PsyCap remains across all industries.

An important contribution of this study however lies in the role of psychopathy which does not fully reflect previous results. While Zhu and Geng [64] found psychopathy to be negatively associated with PsyCap, this study showed that this negative relation does not apply for all industries. Specifically, for architecture, automotive and consulting, the impact does not reach a 5% significance level while it does for the other industries. The highest effect sizes were among public service, health care and education sectors indicating that a more psychopathic personality leads to significantly lower values of hope, efficacy, resilience and optimism. According to other studies, this might result in lower well-being, retention and job success [65]. A potential explanation for industry differences of the relation of psychopathy and PsyCap could be different levels of risk seeking and using structures of prestige that are expected in the sector. For example, jobs in architecture or consulting might require employees to be more tolerant for risk or uncertain situations than in government or education sector. Thus, in the first two industries more personalities might be attracted who are open for taking risks (referred to as homogeneity hypothesis, [27]). Psychopathy trait goes with a lack of fear or even positive affect to fear [34] making them fit better to those expectations and resulting in a lower negative impact on PsyCap facets. In contrast, industries, where psychopathic behavior does not fit well to what is expected, this rather leads to lower levels of resilience or efficacy (PsyCap). To give an example: in the educational sector, there are few uncertainties in the daily work due to a more rigid and less volatile organizational structure. In such an industry, taking risks or utilizing prestige is not expected nor beneficial. If people in this environment still have a higher tendency of traits (e.g., high values in psychopathy), this will result in a mismatch with environment leading to less effective and less resilient perceptions (PsyCap). This study reveals industry differences in psychopathic personalities to initiate a discussion on what the underlying antecedents are. We argue that the trait of psychopathy is harmful for the organizational environment but needs to be seen in context with expectations and the organizational setting. This means that it does not always lead to low levels of psychological resources (PsyCap) for those individuals with psychopathic traits. While for some industries, psychopaths go with lower PsyCap and that might increase workplace deviance, this relation is not as strong in other industries where a better fit with organizational setting occurs. This study adds to examining the role of psychopathy more differentiated based on the industry adding value to previous research which has not taken the context or working environment into account.

As for Machiavellianism, results are inconsistent with previous research that found Machiavellianism as well as psychopathy to negatively affect PsyCap [64]. In this study, no significant relation was found with PsyCap for any of the industries. One explanation could be that Machiavellianism correlates across industries strongly with psychopathy (r = 0.49) in line with other recent studies [14, 66–68] suggesting an overlap of both facets that could have reduced the effect of Machiavellianism besides psychopathy. Additionally, Machiavellianism did not exhibit a large variance across industries (non-significant differences in H1c) which, following the homogeneity hypothesis, could imply that this trait is not assimilated or attracted by specific industries and is thus not relevant for individual’s psychological resources. The inconsistency for Machiavellianism should be looked at in more detail in future studies.

### Practical implications

Psychopathy as DT personality trait is given a special role in its relation to psychological resources (PsyCap) at the workplace. This is not only because of its negative relation to PsyCap and potential negative consequences on the individual’s well-being or retention [65] and especially negative consequences on the colleagues of this individual [18, 69, 70], but also due to its non-uniform effect based on different industry contexts. While industry contexts and expectations are unlikely to be changed, an individual employee with an assumed to be stable personality trait can be supported to choose the industry that the person’s personality will likely fit best. The development of a person-environment-fit measurement that is applicable to the workplace might be one option to support employees finding an industry that will benefit their psychological resources. It should not be forgotten that psychopathy is seen as the ‘darkest" of the three DT facets [71]. This is due to the negative impact of psychopathic behavior at the workplace on colleagues and subordinates [18, 69, 70]. While providing support for individuals with a psychopathic trait in their respective industries by making expectations explicit and helping employees to reflect on their own behavior to limit potential negative consequences of psychopathy, actions should also involve support for their colleagues to cope with the consequences of psychopaths at work. Stewart, Forth and Beaudette [72] found that approach coping styles (e.g., problem-focused) supported growth despite negative experiences.

This study showed differences in the prevalence of narcissism across industries. With multiple research studies that examined the negative consequences of narcissistic personality at the workplace and this additional finding in regard to industry differences, concepts on how to deal with narcissism at the workplace can now be deliberately developed and implemented in those industries where narcissism is most prevalent. As already suggested by Judge et al. [15], results from the current study further stress that such concepts should be applied in industries Automotive, Finance, Consulting and IT. This might range from consciously evaluating this personality trait in recruiting decisions, defining in which teams competition vs. cooperation is needed more [73], to switching to performance evaluation systems that include self-ratings carefully to prevent narcissistic enhanced self-ratings from undermining a fair evaluation for employees [74].

### Future research suggestions

In this study, differences in DT across industries were assumed to come from both choosing an industry that matched individuals’ personalities and an assimilation taking place within the industry to align with its respective expectations. Additionally, most people in each industry have a distinct educational background that could be an antecedent of future industry differences in personality and thus needs to be taken into account. Vedel’s study and other studies discovered differences in DT facets among students of different university majors indicating a socialization already taking place within the young adult stage [47, 75, 76]. Future research should thus have a close look at when an assimilation of personality and context takes place. This could be either through measuring educational background together with current industry sector and personality traits, or through a longitudinal approach to examine the change in personality traits over time (starting as young student, after graduation and 5–10 years in the job).

The study showed that psychopathic traits do not (negatively) relate to PsyCap in a few industries while for all others it relates to less psychological resources and can thus be seen as harmful to the individual at work. The design did not take the impact on others in the individual’s team into account, nor did it measure what this does to a company’s climate. With these results on an individual level only, future research should also incorporate contextual factors on team or company level to understand the (non or negative) impact of psychopathic traits on PsyCap and their wider influence within different industries.

### Limitations

The present study is not without limitations that should be addressed in future research to extend its findings in a cross-industrial setting. First, we only used self-report questionnaires. Some studies point out possible issues of biased answering both in the context of PsyCap [12] and in the context of the DT, e.g., narcissists tending towards overclaiming and overestimation as part of their self-regulation strategy [77–79]. Although this might be less critical in online surveys inducing lower tendencies of social desirability [80], future research might consider the use of implicit measurement instruments, like the *Implicit PsyCap Questionnaire* (I-PCQ, [81]). Furthermore, it could be investigated whether the self-ratings of *DT* are consistent with the judgements of others [15, 82, 83] or biological indicators of elevated PsyCap such as the level of cardiovascular capability for recovery to stressful events or level of cortisol output [12, 84].

Second, the *SD3*, as a shorter measure of the DT, has been criticized for focusing on particular aspects of traits of the DT while neglecting others [13, 85]. One criticism is that the psychopathy subscale overrepresents antisocial behavior and impulsivity while neglecting interpersonal manipulation and callous affect [13]. Another criticism is that the narcissism subscale focuses only on the grandiose subtype while neglecting the vulnerable sides of the personality trait [85]. Just recently, Starlinger, Vorcek and Tran [86] found that even within vulnerable narcissism two distinct factors (egocentricity and sensitivity for judgement) are prevalent and might contribute to organizational outcome. Therefore, future research should seek to replicate the results by controlling for egocentricity and measuring narcissism as a bi-dimensional construct.

Third, this study focused on industry differences using the *homogeneity hypothesis* [27] as theoretical explanation. More research is needed in this field to fully understand the antecedents of industry differences. As mentioned before, socialization due to the choice of a specific university major can be one factor. Analogue to a gap identified by Braun [87], we suggest to explicitly measure perceived person-organization-fit [88] and integrate organizational climate, industry specific requirements of the job or leadership perceptions to measure what role the fit of personality to organization plays. For this, existing research on leader-subordinate relation [17, 18, 69] should be taken into account or at least be controlled for.

## Conclusion

The current study provides evidence of DT personality traits predicting PsyCap perceptions differently depending on the industry. The outstanding factor is psychopathy which relates significantly to PsyCap in most industries but does not for sectors of architecture, automotive and consulting. We propose that the extent of assimilation of a person to organizational expectations (e.g., person-organization-fit) is relevant to explain industry differences as an organizational setting attracts different types of personalities. This could also explain the difference in the impact of DT on PsyCap. The study further shows that narcissism was found to be positively associated with PsyCap as this trait enables individuals to interpret their environment in a more positive way. Contrary, psychopathy is the DT facet that needs to be considered thoroughly due to negative effects on PsyCap that vary depending on the industry. Future research should consider these industry effects and re-evaluate current understandings of DT and PsyCap mechanisms based on the hypothesis of homogeneity.

### Electronic Supplementary Material

Below is the link to the electronic supplementary material

Supplementary Material 1

## Data Availability

The data set, R code and variable overview can be downloaded from the OSF repository https://osf.io/s86fy/.
