# Supplementary material for: Where a psychopathic personality matters at work: a cross-industry study of the relation of dark triad and psychological capital
Source: BMC Psychol. 2023 Aug 17;11:236. doi: 10.1186/s40359-023-01266-4 (PMC10436650; doi:10.1186/s40359-023-01266-4)
Supplement: Supplementary file 1 — Supplementary Material 1 [file 40359_2023_1266_MOESM1_ESM.docx]

Categorization of “other” industry category

*Categorization description*

Participants who could not assign themselves were asked to name their industry in an open field. With this approach, additional 6.6% of the sample could be later manually assigned to existing categories with two senior researchers working together to ensure multiple perspectives while decreasing personal bias as suggested by Corbin and Strauss (46).

*Overview industry sectors*

| mapped open responses | mapped to following industry | #of responses  (% of industry group) | not included responses (examples) |
| --- | --- | --- | --- |
| Building materials Real estate Facility/Building services | Architecture & construction | 10 (9%) |  |
| Aviation industry/technology Automotive services Hydraulics | Automotive & Engineering | 5 (3%) |  |
| Accounting Insurance industry Tax advisor/Auditing | Finance & Insurance services | 6 (2%) |  |
| Legal Consulting Personnel Consulting | Consulting | 4 (3%) |  |
| Research Market research Tutoring Adult/Family education | Education & Research | 14 (10%) | Research and Development |
| Fitness/Sports Chemical/pharmaceutical industry Healthcare/Beauty industry Non profit | Health Care, Medical and Social services | 27 (12%) |  |
| Logistics/transportation/freight Wholesale/Sales/Retail trade Publishing/Media | Retail & Consumption | 48 (17%) |  |
| Traffic Authority Public service Administration | Public Service, Administration & Transportation | 5 (2%) | Aviation industry |
| Chemistry Media/Communication/Marketing/  Advertising Tourism Catering Mechanical Engineering | Others | 305  *(were kept in the category)* |  |

*References*

Corbin J, Strauss A. Basics of qualitative research: Techniques and procedures for developing grounded theory, 3rd ed. Thousand Oaks, CA, US: Sage Publications, Inc; 2008. xv, 379 p. (Basics of qualitative research: Techniques and procedures for developing grounded theory, 3rd ed.).
